# USING QUALITATIVE METHODS TO CULTURALLY ADAPT A COMMUNITY-ENGAGED PHYSICAL ACTIVITY INTERVENTION FOR LATINOS

**DOI:** 10.1093/geroni/igae098.3497

**Published:** 2024-12-31

**Authors:** Zvinka Zlatar, Lazaro Martinez Lujan, Mikael Anne Greenwood-Hickman, Julie Cooper, Stefani Florez Acevedo, Andrea-Paula Vargas, Dori Rosenberg

**Affiliations:** University of California San Diego, La Jolla, California, United States; Kaiser Permanente Washington Health Research Institute, Seattle, Washington, United States; Kaiser Permanente Washington Health Research Institute, Seattle, Washington, United States; Kaiser Permanente Washington Health Research Institute, Kaiser Permanente Washington Health Research Institute, Washington, United States; University of Washington, Seattle, Washington, United States; University of California San Diego, La Jolla, California, United States; Kaiser Permanente Washington Health Research Institute, Seattle, Washington, United States

## Abstract

Hispanics/Latinos are at increased risk of developing Alzheimer’s Disease and related dementias (ADRD), but culturally adapted interventions to prevent ADRD in this high-risk population are scarce. We aimed to develop a community-engaged physical activity (PA) intervention to lower ADRD risk among middle-aged and older Hispanic/Latino adults through qualitative feedback from a community advisory board (CAB) and pilot study participants. Ten Hispanic/Latino community members (average age = 67.2, 90% female, 90% Spanish-speaking) participated in a 3-week pre-post remote PA study. Participants received a Fitbit and two health coaching calls from a bilingual/bicultural health coach. Qualitative data were collected through two focus groups with pilot participants and two CAB meetings (seven members) to inform the culturally adapted intervention. Thematic analysis of transcribed and translated focus groups was conducted. Themes from notes taken at CAB meetings were also compiled. Focus group data themes highlighted that the intervention was well-received, with participants recognizing the cognitive benefits of PA engagement, the significance of prioritizing time to oneself, an increased sense of commitment, and identifying the Fitbit as an effective tool as facilitators. Barriers to engagement included life obligations, ingrained habits, adverse weather, and challenges related to physical and mental health factors. The CAB helped to design the study logo and recruitment materials while pilot participants identified a desire for group activities and face-to-face interaction with study staff. Participant and CAB feedback was instrumental in the cultural adaptation process to refine the intervention. Community-engaged approaches are crucial for ADRD prevention in high-risk Hispanic/Latino older adults.

